# Genetic diversity of whitefly (*Bemisia* spp.) on crop and uncultivated plants in Uganda: implications for the control of this devastating pest species complex in Africa

**DOI:** 10.1007/s10340-021-01355-6

**Published:** 2021-03-10

**Authors:** Habibu Mugerwa, John Colvin, Titus Alicai, Christopher A. Omongo, Richard Kabaalu, Paul Visendi, Peter Sseruwagi, Susan E. Seal

**Affiliations:** 1 Natural Resources Institute, University of Greenwich, Central Avenue, Chatham Maritime, Kent, ME4 4TB UK; 2 Department of Entomology, University of Georgia, 1109 Experiment Street, Griffin, GA 30223 USA; 3 Root Crops Programme, National Crops Resources Research Institute, P. O. Box 7084, Kampala, Uganda; 4 Biotechnology Department, Mikocheni Agricultural Research Institute, P.O. Box 6226, Dar es Salaam, Tanzania; 5 Centre for Agriculture and Bioeconomy, Queensland University of Technology, Brisbane, 4001 Australia

**Keywords:** Whitefly, *Bemisia tabaci*, *MtCO1*, Genetic diversity, Host range, East Africa

## Abstract

Over the past three decades, highly increased whitefly (*Bemisia tabaci*) populations have been observed on the staple food crop cassava in eastern Africa and associated with ensuing viral disease pandemics and food insecurity. Increased whitefly numbers have also been observed in other key agricultural crops and weeds. Factors behind the population surges on different crops and their interrelationships are unclear, although in cassava they have been associated with specific populations within the *Bemisia tabaci* species complex known to infest cassava crops in Africa. This study carried out an in-depth survey to understand the distribution of *B. tabaci* populations infesting crops and uncultivated plant hosts in Uganda, a centre of origin for this pest complex. Whitefly samples were collected from 59 identified plant species and 25 unidentified weeds in a countrywide survey. Identities of 870 individual adult whiteflies were determined through *mitochondrial cytochrome oxidase 1* sequences (651 bp) in the 3′ barcode region used for *B. tabaci* systematics. Sixteen *B. tabaci* and five related whitefly putative species were identified based on > 4.0% nucleotide divergence, of which three are proposed as novel *B. tabaci* putative species and four as novel closely related whitefly species. The most prevalent whiteflies were classified as *B. tabaci* MED-ASL (30.5% of samples), sub-Saharan Africa 1 (SSA1, 22.7%) and Bemisia Uganda1 (12.1%). These species were also indicated to be the most polyphagous occurring on 33, 40 and 25 identified plant species, respectively. Multiple (≥ 3) whitefly species occurred on specific crops (bean, eggplant, pumpkin and tomato) and weeds (*Sida acuta* and *Ocimum gratissimum*). These plants may have increased potential to act as reservoirs for mixed infections of whitefly-vectored viruses. Management of whitefly pest populations in eastern Africa will require an integration of approaches that consider their degree of polyphagy and a climate that enables the continuous presence of crop and uncultivated plant hosts.

**Supplementary Information:**

The online version contains supplementary material available at 10.1007/s10340-021-01355-6.

## Key message

- Partial *mtCO1* sequences of 870 whiteflies from > 59 plant species across Uganda, a country known as a centre of diversity for this pest complex, indicated 16 *Bemisia tabaci* (three of which are novel species) and five closely related species (four of which were novel).
- MED-ASL, SSA1 and B. Uganda1 whiteflies were the most abundant, widely distributed and polyphagous. Control measures targeting these populations will be challenging with the need to consider the wide range of uncultivated plants that may act as refuges.

## Introduction

Production of food, vegetable and cash crops in Uganda over the last two decades has been constrained dramatically by pests and diseases (Nabbumba and Bahiigwa 2003; PARM 2017; Echodu et al. 2019). Whiteflies belonging to the genus *Bemisia* are among the most important pests (Okonya and Kroschel 2016; Gayi et al. 2017; Mbeyagala et al. 2017; Kalyebi et al. 2018). *Bemisia tabaci* species cause direct feeding damage on plants through extraction of large quantities of phloem sap leading to the excretion of honeydew which serves as a medium for sooty mould growth reducing photosynthesis and marketable produce (Byrne and Bellows 1991). Collectively, *B. tabaci* species can vector several hundred viruses, the vast majority (> 320 species) belonging to the genus *Begomovirus*, and other economically important whitefly-transmitted viruses belonging to the genera *Ipomovirus*, *Carlavirus, Crinivirus, Torradovirus* and *Polerovirus* (https://ictv.global/report; Polston et al. 2014; Zerbini et al. 2017; Ghosh et al. 2019). Begomoviruses have for several decades been considered the major group of emerging plant pathogens globally (Varma and Malathi 2003; Seal et al. 2006a, b; García-Arenal and Zerbini 2019). Begomovirus disease outbreaks are commonly associated with increased whitefly abundance that can elevate exchange of viruses within the crop as well as with neighbouring uncultivated plants (García-Arenal and Zerbini 2019).

In sub-Saharan Africa, specifically Uganda, dramatically increased whitefly population densities (> 200 whitefly adults for the top five leaves) (Sseruwagi et al. 2004) were first reported on the staple food crop cassava in 1990 in association with the severe cassava mosaic disease (CMD) pandemic that has caused devastation since this time (Otim-Nape et al. 2000; Colvin et al. 2004). Recombination and reassortments among genome components of begomoviruses causing cassava mosaic, as well as in association with disease outbreaks in other exotic crops, are well documented for the African continent as well as in the neighbouring south-west Indian Ocean Islands (Seal et al. 2006a; Lefeuvre et al. 2007; Rey et al. 2012; Rey and Vanderschuren 2017). Cassava is native to South America (Leone 1977; Olsen and Schaal 2001) and its introduction to West and East Africa is considered to have occurred via the Indian Ocean islands in the eighteenth century (Guthrie 1987). Both cassava whitefly vector populations driving the African cassava mosaic pandemic and the causal viruses are, however, not present in South America and are considered indigenous to eastern Africa (Ndunguru et al. 2005; Rey et al. 2012; Boykin et al. 2013; Mugerwa et al. 2018). A handful of uncultivated plant species in Uganda have been discovered as hosts colonised by cassava *B. tabaci*, but with many of these weeds also non-indigenous to Africa, the native plant host ranges of the African cassava whitefly populations remain elusive (Sseruwagi et al. 2005, 2006).

A number of suggestions have been made as to the causes of the dramatic cassava whitefly population increases over the past few decades, which have included (a) the presence of highly fecund invasive *B. tabaci* species on cassava (Legg et al. 2002, 2014b; Sseruwagi et al. 2005; Boykin et al. 2018) and (b) an increase in the cultivation of virus-resistant but whitefly-susceptible cassava varieties in Uganda that were reported to attract high populations of whitefly (Omongo et al. 2004, 2012). It is apparent that there are many factors contributing to the increased whitefly abundance and that singling out any with higher impact is complex (Macfadyen et al. 2018, 2020). There are many knowledge gaps that still need to be addressed and one of these is to what extent other crops and uncultivated plants contribute to the population dynamics of the abundant whitefly populations on cassava.

Members of the *B*. *tabaci* cryptic species complex possess distinct biological traits not only in their abundance and invasiveness, but also in their resistance to insecticides, host colonisation range, inducement of phytotoxic disorders and ability to transmit specific begomoviruses (Perry 1985; Brown et al. 1995; Jones 2003; Seal et al. 2006b; Liu et al. 2007; Vyskočilová et al. 2019; Chi et al. 2020). In the absence of reliable morphological features, *mtCO1* barcoding (Frohlich et al. 1999; Dinsdale et al. 2010), genome-wide SNP markers and whole genome sequencing approaches (Wosula et al. 2017; Chen et al. 2019; de Moya et al. 2019; Elfekih et al. 2019; Mugerwa et al. 2020) have been used to generate an improved understanding of the systematics within this species complex. A partial region of the *mtCO1* gene has been the molecular marker used most widely by the whitefly research community to classify *B. tabaci* species, with to date > 40 cryptic putative species proposed based on > 3–4% nucleotide divergence (Frohlich et al. 1999; Dinsdale et al. 2010; Mugerwa et al. 2018; Vyskocilova et al. 2018; Kunz et al. 2019). In Africa, *B*. *tabaci* species (East Africa 1 (EA1), Indian Ocean (IO), Mediterranean (MED), Middle East-Asia Minor (MEAM1, MEAM2-Africa), Morocco, New World (NW1)-Sudan [EU760727] and sub-Saharan Africa (SSA) species SSA1‒SSA13 have been reported on beans, cassava, cotton, eggplant, tomato, sweet potato or various uncultivated plants (Frohlich et al. 1999; Legg et al. 2002, 2014b; Berry et al. 2004; Sseruwagi et al. 2006; Boykin et al. 2012; Mugerwa et al. 2012, 2018; Tahiri et al. 2013; Esterhuizen et al. 2013).

Although the 3′ *mtCO1* sequence has been widely used to delimit cryptic species within the *B. tabaci* complex, studies are progressively revealing some classifications to be errors caused by nuclear mitochondrial DNA (NUMTs) or chimeric PCR products (Tay et al. 2017; de Moya et al. 2019; Vyskočilová et al. 2018; Kunz et al., 2019), as well as species status not correlating consistently with divergence in the *mtCO1* barcode region (Qin et al. 2016; Vyskočilová et al. 2018; Mugerwa et al. 2020). For describing whitefly diversity in this study, we have nevertheless adopted it as a method to indicate species which should be considered as putative awaiting further biological and genetic studies to confirm their taxonomic status. We have also within specific species referred to subgroups to assist correlation with the existing literature; within SSA1, five subgroups (SG) have been reported (Legg et al. 2014b; Ghosh et al. 2015) and at least two distinct species are present within SSA1 (Mugerwa et al. 2020). Similarly, within the MED species, a population termed ‘ASL’ has been reported to be a distinct ‘non-MED’ species based on its failure to interbreed with MED-Q1 and MED-Q2 populations (Vyskočilová et al. 2018). In this study, the host range and prevalence of MED-ASL are therefore considered independently of data obtained for MED-Q1 whiteflies.

Field surveys and research efforts on African whiteflies have to date focussed on cassava due to the severity of the viruses they spread able to cause CMD as well as cassava brown streak disease (CBSD) (Storey and Nichols 1938; Monger et al. 2001; Legg et al. 2004, 2006; Pennisi 2010; Alicai et al. 2016; Tomlinson et al. 2017). Increases in whitefly-transmitted disease epidemics and whitefly population densities have, however, been noted in the last decade in eastern Africa across other many other agricultural crops (H. Mugerwa personal observations). In order to understand the factors driving these increases and the interrelationships between whitefly populations on crops and uncultivated plants in eastern Africa, efforts must initially focus on gaining a better understanding of the prevalence and host range of different members of the cryptic species complex *B. tabaci*. The diversity of 121 whiteflies from five weeds surrounding cassava crops in Uganda recently indicated that these were colonised by a staggering 13 different whitefly species (as indicated by *mtCO1* barcode sequence) whose identity and distribution was quite distinct to the diversity reported from cassava (Mugerwa et al. 2018). This study expanded the adult whiteflies characterised to ones collected from 59 identified and 25 unidentified plant species across Uganda to generate a greater understanding of the diversity of whiteflies present on different plant species, as well as reveal possible alternate hosts for whiteflies devastating cassava and other crops in eastern Africa. Such knowledge is vital for the development of effective integrated management practices aimed at controlling the rapidly emerging outbreaks of both whitefly and whitefly-transmitted viral diseases in sub-Saharan Africa.

## Materials and methods

### Field selection and sampling criteria

Whitefly adults were collected on crop and weed plants (Tables 1, 2) in the field from July to August 2013 in Uganda. Sampled sites were selected based on observation of whiteflies on crops and weed species, sampling along the main and rural roads separated by intervals of 10–20 km as described by Sseruwagi et al. (2004) and Mugerwa et al. (2018). Digital photographs of the different weeds were taken to aid host plant species identification. Adult whiteflies were collected using an aspirator and stored in 90% ethanol; adults collected from the same host plant in a sampled site were stored in the same tube, but those collected from different host plants were stored in different tubes. For each collection site, geo-coordinates were recorded using a Geographical Positioning System (GPS, Garmin eTrex Vista Cx) together with the locality name (village, sub-county and district). Geo-coordinates were used to generate maps with ArcGIS 10.3.1 software (http://desktop.arcgis.com/en/).

**Table 1:** Numbers of previously reported *Bemisia tabaci* and ‘non-tabaci’ species collected from cultivated and weed plant species during July–August 2013 in Uganda

Plant scientific name	Common name	Family name	SSA1			SSA2	SSA6	SSA9	SSA10	SSA11	SSA12
			SG1	SG2	SG3						
*Abelmoschus esculentus*	Okra	Malvaceae	0	0	0	0	0	0	0	0	0
*Ageratum conzyoides*	Goatweed	Asteraceae	1	0	0	0	0	2	0	0	0
*Ageratum* sp.	Goatweed-like	Asteraceae	1	1	1	0	0	0	0	0	0
*Amaranthus spinosus*	Pigweed	Amaranthaceae	1	0	0	0	0	0	0	0	0
*Arachis hypogaea*	Peanut	Fabaceae	0	1	0	0	0	0	0	0	0
*Aspilia africana*	Haemorrhage plant	Asteraceae	1	0	0	0	3	0	0	0	0
*Bidens pilosa*	Black jack	Asteraceae	0	1	0	0	0	0	0	0	0
*Bothriocline tomentosa*	Luwelewele	Asteraceae	0	0	0	0	0	0	0	0	0
*Brassica oleracea*	Cabbage	Brassicaceae	0	0	0	0	0	0	0	0	0
*Brassica* sp.	Collard/Sukumawiki	Brassicaceae	0	0	0	0	0	0	0	0	0
*Cannabis sativa*	Marijuana	Cannabiaceae	0	0	0	0	0	0	0	0	0
*Capsicum annuum*	Hot pepper	Solanaceae	1	1	0	0	0	0	0	0	0
*Carica papaya*	Papaya	Caricaceae	0	2	0	0	0	0	0	0	0
*Conyza sumatrensis*	White horseweed	Asteraceae	0	1	0	0	0	0	0	0	0
*Crassocephalum crepidioides*	Fireweed	Asteraceae	0	0	0	0	0	0	0	0	0
*Cucurbita moschata*	Pumpkin	Cucurbitaceae	4	1	1	0	0	3	0	0	0
*Dichrocephala integrifolia*	Bicolor buttonweed	Asteraceae	1	0	0	0	0	0	0	0	0
*Emilia coccinea*	Scarlet tasselflower	Asteraceae	0	0	0	0	0	0	0	0	0
Eputoni (local name)	Eputoni	Unknown	0	0	0	1	0	0	0	0	0
*Erlangea tomentosa*	Ekitokotoko	Asteraceae	0	0	0	0	0	1	0	0	0
*Erythrina abyssinica*	Red hot poker tree	Fabaceae	3	1	0	0	0	0	0	0	0
Etakari (local name)	Etakari	Unknown	0	0	0	0	0	0	0	0	0
*Gossypium herbaceum*	Cotton	Malvaceae	0	0	0	0	0	0	0	0	0
*Helianthus annuus*	Sunflower	Asteraceae	2	0	0	0	0	0	1	0	0
*Hoslundia opposite*	Orange bird berry	Lamiaceae	0	0	1	0	0	1	2	0	0
*Ipomoea batatas*	Sweet potato	Convolvulaceae	1	0	1	0	1	0	0	0	0
*Jatropha gossypifolia*	Bellyache bush	Euphorbiaceae	5	0	0	0	0	0	0	0	0
Kabowabowa (local name)	Kabowabowa	Unknown	1	1	0	0	0	0	0	0	0
Kigombolola (Local name)	Kigombolola	Unknown	0	0	0	0	0	0	0	0	0
*Lantana camara*	Tickberry	Verbenaceae	0	0	0	1	0	0	0	0	0
*Leonotis nepetaefolia*	Lion’s ear	Lamiaceae	0	2	0	0	0	1	0	1	0
Luyamayama (local name)	Luyamayama	Unknown	0	0	0	0	0	1	0	0	0
*Manihot esculenta*	Cassava	Euphorbiaceae	63	6	0	29	0	0	0	0	0
*Manihot glaziovii*	Tree cassava	Euphorbiaceae	1	0	0	0	0	1	0	0	0
*Microglossa pyrifolia*	Kafungankadde	Asteraceae	0	0	0	0	0	0	0	0	0
*Mimosa* sp.	Temba	Fabaceae	1	0	0	0	0	0	0	0	0
*Nicotiana rustica*	Aztec tobacco	Solanaceae	3	0	0	0	0	0	0	0	0
*Nicotiana tabacum*	Tobacco	Solanaceae	0	1	2	0	0	0	0	0	0
Njoka etaruma (local name)	Njoka etaruma	Unknown	0	0	0	0	0	1	0	0	0
*Ocimum gratissimum*	African basil	Lamiaceae	1	0	3	1	70	0	0	0	0
*Oxygonum sinuatum*	Kafumitabagenda	Polygonaceae	0	1	0	0	0	0	0	0	0
*Phaseolus vulgaris*	Common bean	Fabaceae	11	1	5	1	0	1	1	0	0
*Ribes uva-crispa*	Gooseberry	Grossulariaceae	0	0	0	0	0	0	0	0	0
*Rotheca myricoides*	Butterfly bush	Lamiaceae	1	0	0	0	0	2	0	0	0
*Senna occidentalis*	Coffee senna	Fabaceae	0	0	0	0	0	0	0	0	0
*Senna* sp.	Magele ga nkoko	Fabaceae	1	0	0	0	0	0	0	0	0
*Sesamum indicum*	Sesame	Pedaliaceae	2	0	0	0	1	0	0	0	0
*Sida acuta*	Wireweed	Malvaceae	0	3	3	0	1	2	0	0	0
*Solanum aethiopicum*	Bitter tomato	Solanaceae	1	0	0	0	0	0	0	0	0
*Solanum incanum*	Nightshade	Solanaceae	2	0	0	0	0	0	0	0	0
*Solanum lycopersicum*	Tomato	Solanaceae	4	2	0	2	1	6	0	0	0
*Solanum melongena*	Eggplant	Solanaceae	1	0	5	0	0	3	1	1	0
*Solanum nigrum*	Black nightshade	Solanaceae	0	0	0	0	0	0	0	0	0
*Spathodea campanulata*	African tuliptree	Bignoniaceae	0	1	0	0	0	0	0	0	0
*Striga hermonthica*	Giant witchweed	Orobanchaceae	1	0	0	1	0	0	0	0	0
*Tithonia diversifolia*	Tree marigold	Asteraceae	0	0	2	0	2	0	0	0	0
Toovu (local name)	Toovu	Unknown	1	2	0	0	0	0	0	0	0
*Vigna unguiculata*	Cowpea	Fabaceae	4	1	0	0	0	0	1	0	0
Unknowns	Unknowns	Unknown	17	2	5	0	2	2	3	0	2
Total			137	32	29	36	81	27	9	2	2

Plant scientific name	Common name	Family name	SSA13	MED		MEAM1	MEAM2	IO	EA1	B. Uganda1	Total
				ASL	Q1						
*Abelmoschus esculentus*	Okra	Malvaceae	0	6	0	0	0	0	0	0	6
*Ageratum conzyoides*	Goatweed	Asteraceae	0	0	0	0	1	0	0	0	4
*Ageratum* sp.	Goatweed-like	Asteraceae	0	0	0	0	0	0	0	0	3
*Amaranthus spinosus*	Pigweed	Amaranthaceae	0	1	0	1	0	0	0	0	3
*Arachis hypogaea*	Peanut	Fabaceae	0	2	0	0	0	0	0	1	4
*Aspilia africana*	Haemorrhage plant	Asteraceae	0	7	0	1	0	0	0	1	13
*Bidens pilosa*	Black jack	Asteraceae	0	1	0	1	0	0	0	3	6
*Bothriocline tomentosa*	Luwelewele	Asteraceae	0	0	0	0	0	1	0	1	2
*Brassica oleracea*	Cabbage	Brassicaceae	0	3	0	3	0	0	0	2	8
*Brassica* sp.	Collard/Sukumawiki	Brassicaceae	0	0	0	3	0	0	0	0	3
*Cannabis sativa*	Marijuana	Cannabiaceae	0	1	0	0	0	0	0	0	1
*Capsicum annuum*	Hot pepper	Solanaceae	0	0	0	1	0	0	0	0	3
*Carica papaya*	Papaya	Caricaceae	0	0	1	0	0	1	0	2	6
*Conyza sumatrensis*	White horseweed	Asteraceae	0	0	0	0	0	0	0	5	6
*Crassocephalum crepidioides*	Fireweed	Asteraceae	0	0	0	0	0	0	0	1	1
*Cucurbita moschata*	Pumpkin	Cucurbitaceae	1	55	0	2	0	2	1	0	70
*Dichrocephala integrifolia*	Bicolor buttonweed	Asteraceae	0	1	0	0	0	0	0	0	2
*Emilia coccinea*	Scarlet tasselflower	Asteraceae	0	0	0	0	0	0	0	2	2
Eputoni (local name)	Eputoni	Unknown	0	1	2	0	0	0	0	0	4
*Erlangea tomentosa*	Ekitokotoko	Asteraceae	0	1	0	0	0	0	0	0	2
*Erythrina abyssinica*	Red hot poker tree	Fabaceae	0	1	0	0	0	0	0	0	5
Etakari (local name)	Etakari	Unknown	0	3	0	0	0	0	0	0	3
*Gossypium herbaceum*	Cotton	Malvaceae	0	2	0	0	0	0	0	1	3
*Helianthus annuus*	Sunflower	Asteraceae	0	6	0	0	0	0	0	0	9
*Hoslundia opposite*	Orange bird berry	Lamiaceae	0	4	0	0	0	0	0	0	8
*Ipomoea batatas*	Sweet potato	Convolvulaceae	0	38	0	0	0	0	1	37	79
*Jatropha gossypifolia*	Bellyache bush	Euphorbiaceae	0	0	0	0	0	0	0	0	5
Kabowabowa (local name)	Kabowabowa	Unknown	0	0	0	0	0	0	0	0	2
Kigombolola (Local name)	Kigombolola	Unknown	3	0	0	0	0	0	0	0	3
*Lantana camara*	Tickberry	Verbenaceae	0	0	0	0	0	1	0	0	2
*Leonotis nepetaefolia*	Lion’s ear	Lamiaceae	1	0	0	0	0	0	0	0	5
Luyamayama (local name)	Luyamayama	Unknown	0	0	0	0	0	0	0	1	2
*Manihot esculenta*	Cassava	Euphorbiaceae	0	0	0	0	0	0	0	0	98
*Manihot glaziovii*	Tree cassava	Euphorbiaceae	0	0	0	0	0	0	0	1	3
*Microglossa pyrifolia*	Kafungankadde	Asteraceae	0	1	0	0	0	0	0	0	1
*Mimosa* sp.	Temba	Fabaceae	1	0	0	0	0	0	0	0	2
*Nicotiana rustica*	Aztec tobacco	Solanaceae	0	0	0	0	0	0	0	0	3
*Nicotiana tabacum*	Tobacco	Solanaceae	0	1	26	2	0	0	0	3	35
Njoka etaruma (local name)	Njoka etaruma	Unknown	0	0	0	0	0	0	0	2	3
*Ocimum gratissimum*	African basil	Lamiaceae	2	8	0	0	0	0	0	0	85
*Oxygonum sinuatum*	Kafumitabagenda	Polygonaceae	0	0	0	0	0	0	0	2	3
*Phaseolus vulgaris*	Common bean	Fabaceae	1	6	0	0	0	2	1	11	41
*Ribes uva-crispa*	Gooseberry	Grossulariaceae	2	1	0	0	0	1	0	2	6
*Rotheca myricoides*	Butterfly bush	Lamiaceae	0	0	0	0	0	0	0	0	3
*Senna occidentalis*	Coffee senna	Fabaceae	0	2	0	0	0	0	0	0	2
*Senna* sp.	Magele ga nkoko	Fabaceae	0	2	0	0	0	0	0	0	3
*Sesamum indicum*	Sesame	Pedaliaceae	0	8	0	0	0	0	0	1	12
*Sida acuta*	Wireweed	Malvaceae	3	48	6	1	0	1	0	2	70
*Solanum aethiopicum*	Bitter tomato	Solanaceae	0	1	0	0	0	0	0	1	3
*Solanum incanum*	Nightshade	Solanaceae	0	5	1	1	0	0	0	0	9
*Solanum lycopersicum*	Tomato	Solanaceae	2	14	1	7	0	3	1	8	51
*Solanum melongena*	Eggplant	Solanaceae	2	9	1	10	1	1	0	3	38
*Solanum nigrum*	Black nightshade	Solanaceae	2	3	0	0	0	0	0	3	8
*Spathodea campanulata*	African tuliptree	Bignoniaceae	0	1	0	0	0	1	0	0	3
*Striga hermonthica*	Giant witchweed	Orobanchaceae	0	0	0	0	0	1	0	0	3
*Tithonia diversifolia*	Tree marigold	Asteraceae	0	0	0	0	0	0	0	0	4
Toovu (local name)	Toovu	Unknown	0	0	0	0	0	0	0	0	3
*Vigna unguiculata*	Cowpea	Fabaceae	0	0	0	0	0	0	0	0	6
Unknowns	Unknowns	Unknown	4	22	9	6	0	1	0	9	84
Total			24	265	47	39	2	16	4	105	857

Abbreviations for the whitefly species: SSA = sub-Saharan Africa, SG = subgroup, MED = Mediterranean, ASL = African silver leafing, MEAM = Middle East-Asia Minor, IO = Indian Ocean, EA1 = East Africa 1 and B. Uganda = *Bemisia* Uganda

**Table 2:** Numbers of new *Bemisia tabaci* and ‘non-tabaci’ species collected from cultivated and weed plant species during July–August 2013 in Uganda

Plant scientific name	Common name	Plant family name	SSA14	SSA15	SSA16	B. Uganda2	B. Uganda3	B. Uganda4	B. Uganda5	Total
*Bothriocline tomentosa*	Luwelewele	Asteraceae	0	0	1	0	0	0	0	1
*Cucurbita moschata*	Pumpkin	Cucurbitaceae	0	1	0	0	0	0	0	1
*Dichrocephala integrifolia*	Bicolor buttonweed	Asteraceae	0	0	0	0	0	0	1	1
*Erythrina abyssinica*	Red hot poker tree	Fabaceae	0	0	0	0	1	0	0	1
*Hoslundia opposite*	Orange bird berry	Lamiaceae	0	0	0	0	0	1	0	1
*Ipomoea batatas*	Sweet potato	Convolvulaceae	0	0	0	0	0	1	0	1
Kabowabowa (local name)	Kabowabowa	Unknown	0	1	0	0	0	0	0	1
*Leonotis nepetaefolia*	Lion’s ear	Lamiaceae	0	0	1	0	0	0	0	1
*Phaseolus vulgaris*	Common bean	Fabaceae	0	0	0	1	2	0	0	3
*Solanum incanum*	Nightshade	Solanaceae	1	0	0	0	0	0	0	1
Unknown	Unknown	Unknown	0	0	0	0	1	0	0	1
Total			1	2	2	1	4	2	1	13

Abbreviations for the whitefly species: SSA = sub-Saharan Africa and B. Uganda = *Bemisia* Uganda

### Whitefly DNA extraction

Three individual adult whiteflies were selected randomly from each sample. Genomic DNA was extracted from an individual whitefly by crushing it in 50 µL of 10% (w/v) Chelex 100 sodium form solution (Sigma-Aldrich, St Louis, MO, USA) in a 1.5-mL Eppendorf tube using a plastic rod following the procedure of Walsh et al. (1991). The extracts were incubated 20 min at 56 °C, then 5 min at 100 °C before centrifugation (5 min, ~ 16000* g*) and storage at −20 °C till use as template for PCR amplification.

### Mitochondrial DNA amplification, cloning and sequencing

Amplification of the partial *mtCO1* fragment was performed using forward primer 2195Bt (5ʹ-TGRTTTTTTGGTCATCCRGAAGT-3ʹ) and reverse primer C012/Bt-sh2 (5ʹ-TTTACTGCACTTTCTGCC-3ʹ) (Mugerwa et al. 2018). PCR reaction mixtures (20µL) contained 10µL of 2 × reSource™ Taq Mix (reSource Taq DNA Polymerase, 6 mM MgCl_2_, 2 mM dNTPs) (Source BioScience, UK), 1µL of each 10 µM primer, 6µL of molecular biology-grade water (Sigma-Aldrich) and 2µL of DNA template. Initial denaturation (94 °C 2 min) was followed by 35 cycles of denaturation (94 °C, 20 s), primer annealing (52 °C, 30 s) and extension (72 °C, 1 min). A final extension (72 °C, 10 min) was performed before storing reactions at 4 °C. Electrophoresis of PCR products was on 2%(w/v) agarose gels in 0.5 × TBE stained with RedSafe™ (iNtRON Biotechnology, Korea). PCR products were visualised under UV light (302 nm) and those of the expected size (864 bp) purified for sequencing and cloning using a reSource™ PCR purification kit (Source BioScience, UK). Purified PCR products were sent for Sanger sequencing (Source BioScience, UK). Where a novel sequence was identified, purified PCR products were cloned from three separate PCR reactions using the pGEM®-T easy vector kit (Promega, UK) and resequenced to confirm the novel sequence. Sequences generated were deposited in GenBank (accession numbers MK444227-MK445130).

### Identification of NUMTS and chimeric PCR products in generated sequence data

Identification of NUMTs and PCR artefacts in the sequences obtained was as described by Vyskočilová et al. (2018) and Kunz et al. (2019). Briefly, Sanger sequences generated in this study were aligned with high-throughput sequencing (HTS)-derived full mitogenome sequences downloaded directly from GenBank in Geneious Prime® 2019.2.1 with the MUSCLE alignment option set to eight iterations. All Sanger sequences which contained indels were eliminated and not considered for further analysis. The remaining Sanger sequences together with HTS-sequences were then trimmed to 651 bp and translated to amino acid residues from appropriate codon positions using the invertebrate mitochondrial DNA genetic codes to: (i) identify potential premature stop codons and (ii) enable amino acid residue alignment against the HTS reference COI amino acid dataset. Sanger sequences which had premature stop codons and amino acid substitutions in highly conserved regions as identified within the trimmed HTS reference CO1 gene set were eliminated. The remaining sequences (*n* = 870) were used for further analysis.

### Global B. tabaci samples, outgroups and phylogenetic analysis

Whitefly *mtCO1* sequences obtained in this study were aligned together with equivalent reference whitefly sequences obtained from Kunz et al. (2019) in Geneious Prime® 2019.2.1. The model of molecular evolution was determined using JModelTest version 2.1.10. and phylogenetic trees generated using MrBayes version 3.2.6 set with the following commands: lset nst = 6 rates = gamma. MrBayes was run for 50 million generations and trees were sampled every 1000 generations. All runs reached a plateau in likelihood score (i.e. stationarity), which was indicated by the standard deviation of split frequencies (0.015), and the potential scale reduction factor (PSRF) was close to one, indicating the MCMC chains converged. The generated tree file was viewed and edited in FigTree version 1.4.4 (http://tree.bio.ed.ac.uk/software/figtree/).

### Hierarchical analysis of whitefly species present on different plants

Hierarchical cluster analysis was used to infer whitefly–host range profiles based on whitefly numbers on different host plants. Data from Mugerwa et al. (2018) obtained from the same locations and time period were added to the data generated in this study to increase the robustness of the analysis, resulting in 991 sequences from 64 hosts. Host plants on which adult whiteflies were absent were denoted as 0. To find the optimal number of clusters, a combination of 23 cluster validation indices implemented in the R statistical package NbClust (Charrad et al. 2014) were used. Cluster uncertainty was determined using the R package Pvclust (Suzuki and Shimodaira 2006). Clusters with Approximately Unbiased (AU) *p*-values > 83 were considered strongly supported by the data.

## Results

### Sampling and phylogenetic analysis

Three individual whiteflies were extracted and sequenced for each specific host location sample and 39.7% of samples contained a mix of whitefly species. A total of 870 *mtCO1* high-quality sequences from individual whiteflies were selected for further analysis after removing 34 sequences that contained errors/pseudogenes as described in Vyskočilová et al. (2018). The identities of individual whiteflies were determined based on their phylogenetic placement and sequence identity of their partial *mtCO1* sequences with already defined species (Tables 1, 2); sequences that clustered with *B. tabaci* species and diverged < 4.0% from the *mtCO1* nucleotide (nt) sequences of already defined species were classified as the corresponding species. Sequences that diverged by ≥ 4.0% from the *mtCO1* nt sequences of already defined *B. tabaci* species were classified as novel species. Based on these criteria, 16 *B. tabaci* species were identified, of which three were novel (hereby named SSA14-SSA16) as they only shared a maximum nt identity of 86.0‒95.5% to *B. tabaci* sequences already present in GenBank (Table 3). A further five whitefly species were identified that grouped outside but close to the *B. tabaci* species complex. One of these represented *Bemisia* Uganda1 (*n* = 105), while the other four represented novel putative species and only possessed maximum sequence identity of 86.9–88.1% with an unidentified *Bemisia* species (PDBI – MN056066). For the purpose of this manuscript, the new species are referred to as B. Uganda2 (*n* = 1), B. Uganda3 (*n* = 3), B. Uganda4 (*n* = 2) and B. Uganda5 (*n* = 1).

**Table 3:** Estimate of evolutionary divergence (expressed as percent nucleotide identity) between partial mitochondrial cytochrome oxidase I sequence representatives of *Bemisia tabaci* identified on various crops in Uganda as conducted using Tajima-Nei model in MEGA X

		1	2	3	4	5	6	7	8	9	10	11	12	13	14	15	16	17	18	19	20	21	22	23	24	25	26	27	28
1	MED_Q1_UG361b	–																											
2	MED_ASL_UG360b	97.4	–																										
3	MEAM2_Africa_UG345	95.6	95.6	–																									
4	EA1_UG192c	95.1	94.7	95.5	–																								
5	MEAM1_UG347c	94.8	95.0	95.8	94.3	–																							
6	SSA13_UG168bs	93.9	93.3	93.6	93.8	93.8	–																						
7	SSA12_UG245	93.6	93.9	93.8	93.3	93.4	94.5	–																					
8	IO_UG321c	93.5	93.3	93.3	92.8	92.7	92.4	92.8	–																				
9	SSA11_UG101b	82.5	82.3	81.5	83.5	81.7	82.8	82.6	82.4	–																			
10	SSA10_UG56b	83.9	84.6	84.2	84.8	84.8	83.8	85.6	85.0	85.5	–																		
11	NewWorld1_DQ130053	83.5	82.7	83.8	83.1	83.7	81.9	83.8	82.7	81.8	85.1	–																	
12	NewWorld2_JN689355	81.7	81.7	82.7	82.4	82.9	81.9	82.9	82.9	81.5	84.9	95.3	–																
13	SSA15_UG247b	82.9	82.5	83.0	82.8	84.3	82.7	83.9	83.4	82.6	87.8	84.9	83.4	–															
14	SSA14_UG62b	81.2	80.4	80.6	80.3	82.1	80.1	81.5	81.7	82.2	86.0	83.2	81.3	92.9	–														
15	Uganda1_Beans	78.9	79.1	79.7	78.5	79.5	78.7	78.6	78.6	79.3	78.8	80.0	78.2	79.4	78.3	–													
16	BemisiaUganda2_UG203	79.0	78.8	79.5	77.1	79.7	78.6	79.6	78.8	77.0	79.0	78.9	77.7	78.8	77.3	88.2	–												
17	BemisiaUganda3_UG292c	83.6	83.0	84.9	82.6	84.5	84.1	84.3	81.8	79.9	82.1	82.9	80.3	80.3	78.0	84.4	86.0	–											
18	BemisiaspPDB1_MN056066	83.3	83.3	83.6	84.1	84.2	84.4	85.0	82.7	82.2	84.3	83.0	81.9	83.6	81.7	85.0	87.4	88.8	–										
19	BemisiaUganda4_UG175b	80.0	80.4	80.0	79.5	80.2	82.3	80.3	80.0	77.2	81.5	79.9	80.1	82.4	80.2	81.4	83.3	83.0	86.9	–									
20	BemisiaUganda5_UG141	83.9	82.9	84.0	83.1	84.6	83.7	84.3	83.3	80.1	85.1	83.7	82.7	82.8	80.6	83.4	84.9	86.2	88.1	87.7	–								
21	SSA1-SG1_UG22c	81.2	80.8	82.2	81.0	82.1	81.6	81.6	79.7	79.3	81.5	82.5	80.5	82.0	80.3	79.2	81.9	84.7	83.5	83.9	84.1	–							
22	SSA1-SG2_UG200	81.4	81.4	82.3	81.2	82.3	81.6	81.6	79.9	79.2	82.5	82.2	80.8	81.8	79.3	79.0	82.0	84.4	83.9	84.3	83.9	98.5	–						
23	SSA1-SG3_UG10	81.2	80.4	82.1	81.0	81.7	81.4	81.8	79.7	79.0	81.7	82.8	80.8	81.6	79.5	79.6	81.8	84.8	83.7	84.1	84.3	99.1	98.8	–					
24	SSA6_UG303b	80.7	80.1	81.7	81.0	81.3	81.8	82.0	81.5	81.1	81.8	82.7	81.1	80.8	78.9	79.0	81.3	81.7	82.6	84.7	83.8	92.4	91.9	92.3	–				
25	SSA3_KM377923	81.4	80.2	81.4	82.2	82.2	81.6	82.0	81.6	80.9	81.6	83.5	81.8	82.8	80.2	79.1	82.1	83.5	84.0	83.8	83.9	92.4	91.6	91.9	92.2	–			
26	SSA2_UG143c	81.0	80.6	81.6	80.2	82.2	82.0	81.6	81.2	80.5	82.5	83.6	81.4	81.0	78.7	79.4	82.8	85.9	83.3	84.3	84.2	91.7	91.2	91.2	90.5	93.7	–		
27	SSA9_UG151c	81.7	81.1	80.7	82.4	81.7	81.5	82.1	81.8	80.1	81.4	83.3	82.1	80.1	77.4	78.5	80.1	83.2	82.4	82.8	85.3	92.3	91.1	92.1	91.7	93.5	92.8	–	
28	SSA16_UG99	80.4	79.6	80.1	81.4	81.6	81.5	81.5	81.2	80.9	81.9	83.7	82.8	81.0	78.3	79.5	82.5	84.1	84.5	83.4	84.6	92.6	91.7	92.1	91.9	93.8	93.8	95.5	–

Reference sequences obtained from GenBank were used for NewWorld1, NewWorld2, *Bemisia* sp.PDB1 and SSA3

**Fig. 1:**
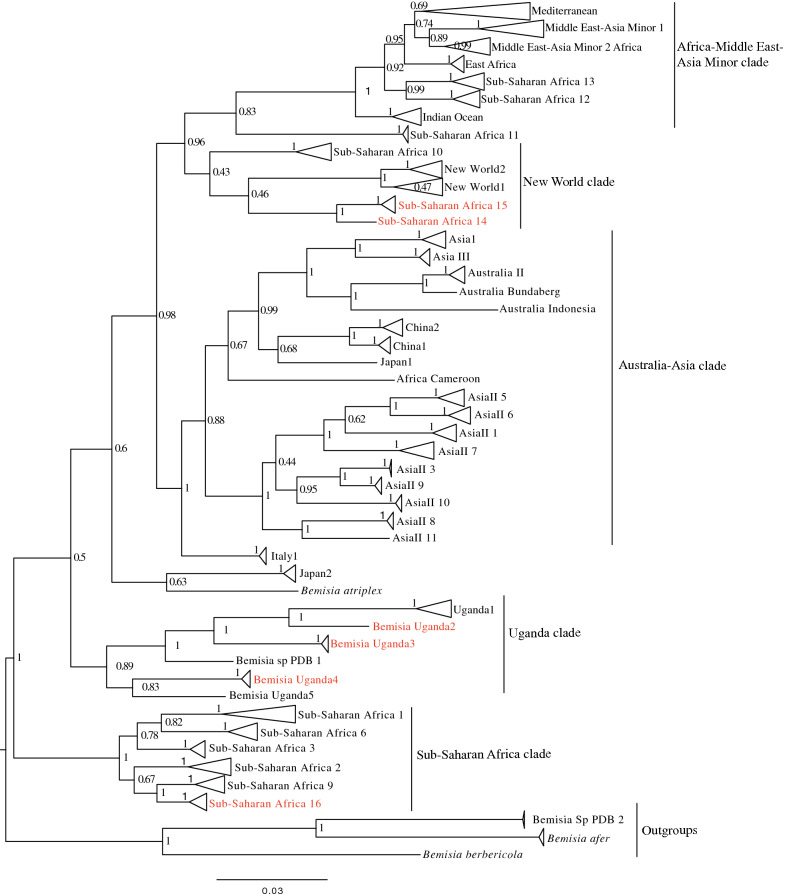
Phylogeny for *Bemisia tabaci* based on partial *mtCO1* sequences. In red font are the newly identified whitefly putative species. Numbers on nodes are probability values

Phylogenetic analysis (Fig. 1) grouped the partial *mtCO1* sequences into four of the 11 high-level genetic groups (HLGG) identified by Dinsdale et al. (2010) and termed ‘Uganda’, ‘SSA’, ‘New World’ and ‘Africa-Middle East-Asia Minor’. A phylogeny with collapsed branches is presented in Fig. 1, while a phylogeny with un-collapsed branches is presented in Supplementary Fig. 1. At the base of the phylogeny, six SSA species are placed in the SSA HLGG with a probability value of 1, separating them from the six species in the Uganda clade (B. Uganda1-5 and *Bemisia* sp. PDB1). Within the SSA HLGG, our study identified 344 sequences assigned to SSA1 (*n* = 198), SSA2 (*n* = 36), SSA6 (*n* = 81) and SSA9 (*n* = 27), as well two whiteflies representing a new putative species SSA16 (Fig. 1). No representatives of SSA3 were found. The novel SSA16 sequences had 95.5% sequence identity to an SSA9 sequence (UG99) identified in this study.

The New World HLGG had a probability value of 0.96 and consisted of five clades with the sequences obtained previously from the New World forming two of the clades clustering together but away from the SSA species. Adjacent to NW1 and NW2 species were SSA14 and SSA15 supported with a low probability value of 0.46, while SSA10 species was indicated to be basal in the New World HLGG, but with a low probability value of 0.43. SSA14 and SSA15 shared 81.3–84.9% sequence similarity with New World 1 and 2 species (Table 3).

A large proportion (46%) of the Ugandan whitefly sequences from this study clustered into the Africa-Middle East-Asia Minor HLGG clade, distinct from the other clades with 0.96 probability value. The 401 partial 651 bp *mtCO1* ‘barcode’ sequences were assigned to previously described species as follows: EA1 (*n* = 4), IO (*n* = 16), MEAM1 (*n* = 39), MEAM2 Africa (*n* = 2), MED-ASL (*n* = 265), MED-Q1 (*n* = 47), SSA11 (*n* = 2), SSA12 (*n* = 2) and SSA13 (*n* = 24).

### Abundance and host range of whitefly species

**Fig. 2:**
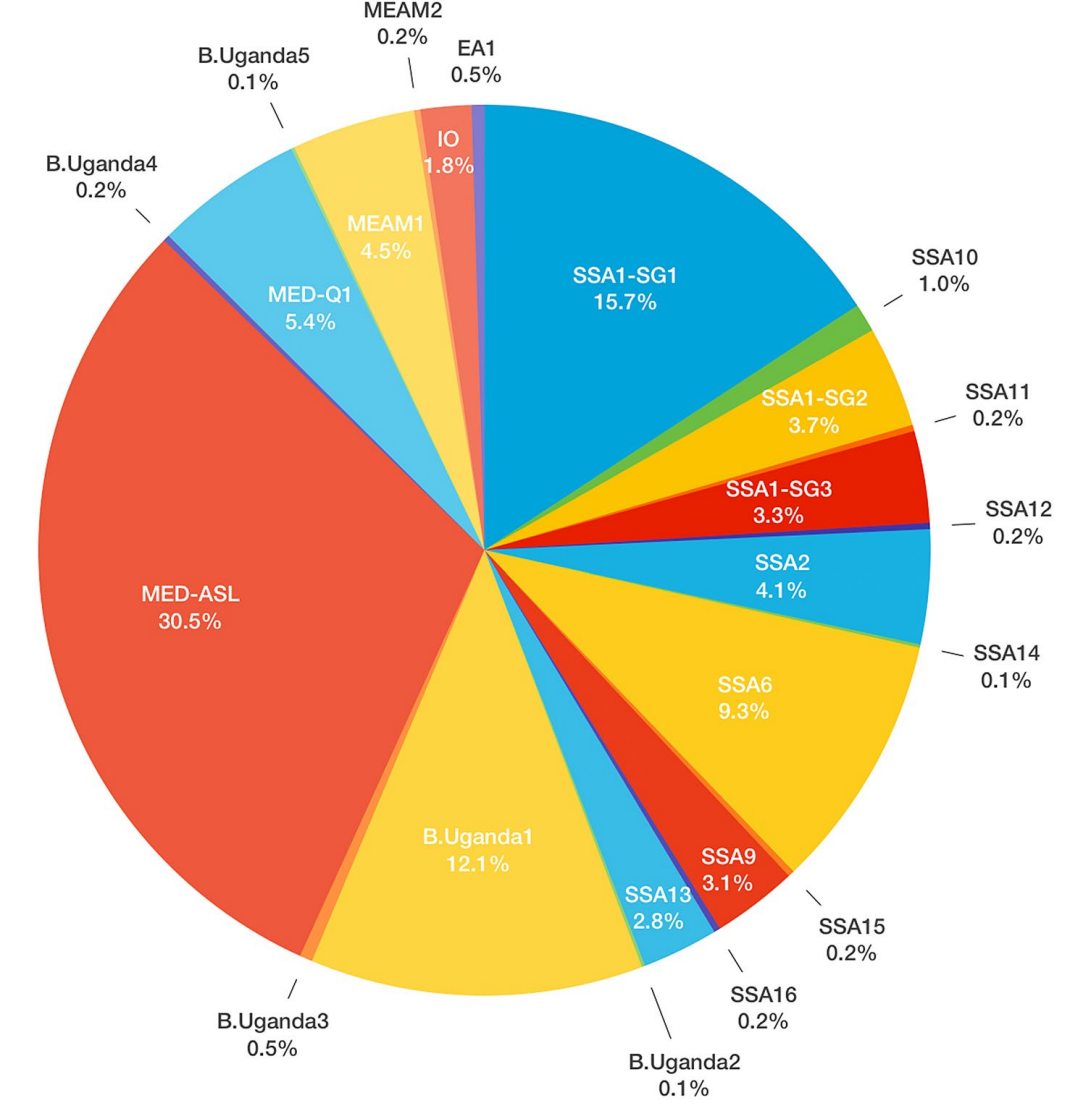
Percentage composition of whitefly species identified in Uganda on different host plants during July–August 2013. The abbreviations for the species: SSA = sub-Saharan Africa, IO = Indian Ocean, MEAM = Middle East-Asia Minor, MED = Mediterranean and B. Uganda = Bemisia Uganda

**Fig. 3:**
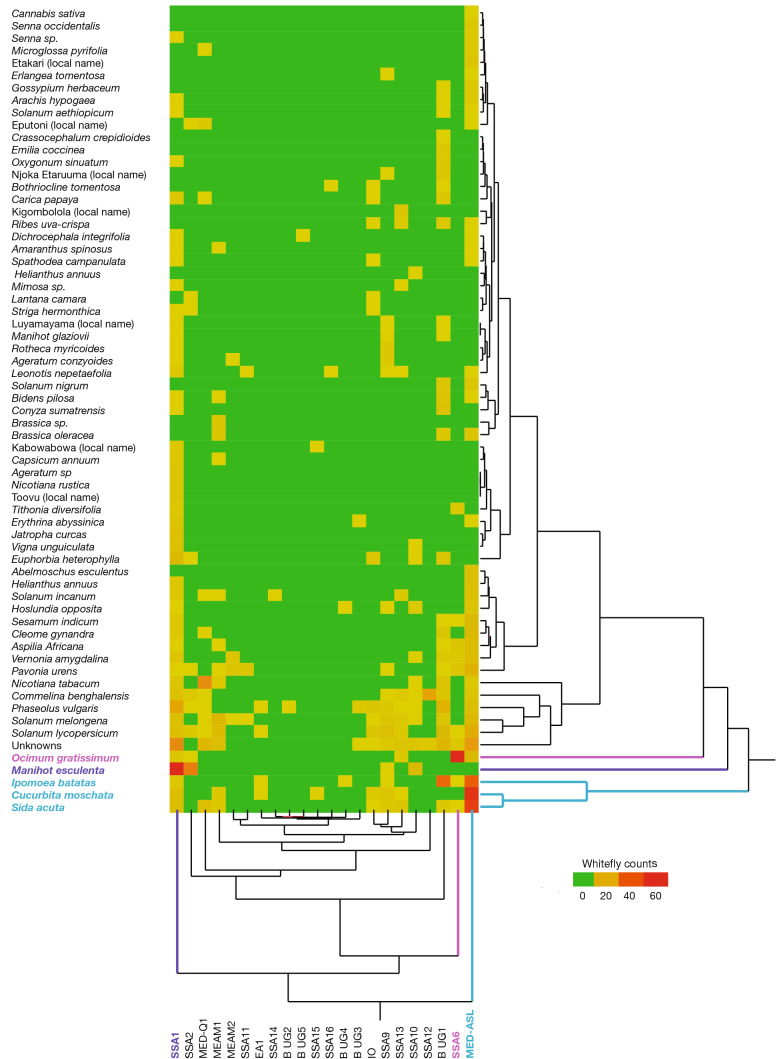
Hierarchical clustering of whitefly numbers on host plants. Four clusters of host plants (Y axis) and whitefly species (X axis) were observed. Host plants clusters are denoted by black, pink, purple and cyan correspond to the whitefly clusters with the same colours on the X axis. SSA1 (purple), SSA6 (pink) and MED-ASL (cyan) have distinct host profiles on *Manihot esculenta* (purple), *Ocimum gratissimum* (pink) and *Ipomoea batatas, Cucurbita moschata, Sida acuta* (cyan), respectively

The most abundant whitefly species identified from individual *mtCO1* sequences were MED-ASL (30.5%), SSA1 (SG1 = 15.7%, SG2 = 3.7% and SG3 = 3.3; 22.7%) and B. Uganda1 (12.1%) (Fig. 2). These species were also found on the largest number of different identified plant species (33 for MED-ASL, 40 for SSA1 and 25 for B. Uganda1), but the presence of a small number of flies on a host can be through chance particularly for more abundant species. Hierarchical clustering of whitefly species numbers based on their presence on the various host plants was therefore undertaken and revealed four distinct clusters based on optimal cluster numbers estimated using NbClust (Fig. 3, Supplementary Fig. 2). MED-ASL, SSA1 and SSA6 formed separate individual clusters with MED-ASL predominantly found on *Cucurbita moschata* (pumpkin, 55/265 whiteflies), *Sida acuta* (wireweed, 48/265) and *Ipomoea batatus* (sweet potato, 38/265), whereas SSA1 was predominantly found on *Manihot esculenta* (cassava, 69/198) and SSA6 on *Ocimum gratissimum* (African basil, 70/81). The fourth cluster consisted generally of low and variable numbers of whiteflies on the other plant hosts (Supplementary Fig. 2), but with a sub-cluster of B. Uganda1, MED Q1 and SSA2 due to their strong associations with sweet potato (37/105 B. Uganda1), tobacco (26/47 MED Q1) and cassava (29/36 SSA2).

Hierarchical clustering of host plants based on whiteflies detected was also performed. Four groups were apparent with cassava and African basil-forming individual clusters due to each having exceptionally high numbers of SSA1-SSA2 and SSA6 whiteflies, respectively. A third cluster consisted of sweet potato*, Sida acuta* and pumpkin which all had high numbers of MED-ASL whiteflies (Supplementary Fig. 3). The fourth cluster consisted of the rest of the host plants which had lower numbers of whiteflies and less robust associations.

The data was also examined by eye to determine whether further whitefly–host plant species associations were suggested. If associations supported by at least three typed whiteflies are selected, a pattern of multiple whitefly species found on specific crops becomes apparent (Table 4). *B. tabaci* species SSA1 and SSA2 are known as ‘cassava whiteflies’ and this close host association holds for SSA2, but SSA1 whiteflies appeared more polyphagous with ≥ 3 whiteflies collected from bean, cowpea, eggplant, *Jatropha gossypifolia, N. rustica*, pumpkin and tomato as well as the weeds *Erythrina abyssinica, O. gratissimum* and *S. acuta*.

**Table 4:** Predominant *Bemisia tabaci* and Bemisia Uganda1 associations with plants from which collections were made in 2013 survey

Plant scientific name	Common name	Family name	SSA1			SSA2	SSA6	SSA9	SSA13	MED		MEAM1	IO	B. Uganda1	Total wfs
			SG1	SG2	SG3					ASL	Q1				
*Abelmoschus esculentus*	Okra	Malvaceae	0	0	0	0	0	0	0	**6**	0	0	0	0	6
*Aspilia Africana*	Haemorrhage plant	Asteraceae	1	0	0	0	**3**	0	0	**7**	0	1	0	1	13
*Bidens Pilosa*	Black jack	Asteraceae	0	1	0	0	0	0	0	1	0	1	0	**3**	6
*Brassica oleracea*	Cabbage	Brassicaceae	0	0	0	0	0	0	0	**3**	0	**3**	0	2	8
*Brassica* sp.	Collard/Sukumawiki	Brassicaceae	0	0	0	0	0	0	0	0	0	**3**	0	0	3
*Conyza sumatrensis*	White horseweed	Asteraceae	0	1	0	0	0	0	0	0	0	0	0	**5**	6
*Cucurbita moschata*	Pumpkin	Cucurbitaceae	**4**	1	1	0	0	**3**	1	**55**	0	2	2	0	69
*Erythrina abyssinica*	Red hot poker tree	Fabaceae	**3**	1	0	0	0	0	0	1	0	0	0	0	5
Etakari (local name)	Etakari	Unknown	0	0	0	0	0	0	0	**3**	0	0	0	0	3
*Helianthus annuus*	Sunflower	Asteraceae	2	0	0	0	0	0	0	**6**	0	0	0	0	8
*Hoslundia opposite*	Orange bird berry	Lamiaceae	0	0	1	0	0	1	0	**4**	0	0	0	0	6
*Ipomoea batatas*	Sweet potato	Convolvulaceae	1	0	1	0	1	0	0	**38**	0	0	0	**37**	78
*Jatropha gossypifolia*	Bellyache bush	Euphorbiaceae	**5**	0	0	0	0	0	0	0	0	0	0	0	5
Kigombolola (Local name)	Kigombolola	Unknown	0	0	0	0	0	0	**3**	0	0	0	0	0	3
*Manihot esculenta*	Cassava	Euphorbiaceae	**63**	**6**	0	**29**	0	0	0	0	0	0	0	0	98
*Nicotiana rustica*	Aztec tobacco	Solanaceae	**3**	0	0	0	0	0	0	0	0	0	0	0	3
*Nicotiana tabacum*	Tobacco	Solanaceae	0	1	2	0	0	0	0	1	**26**	2	0	**3**	35
*Ocimum gratissimum*	African basil	Lamiaceae	1	0	**3**	1	**70**	0	2	**8**	0	0	0	0	85
*Phaseolus vulgaris*	Common bean	Fabaceae	**11**	1	**5**	1	0	1	1	**6**	0	0	**2**	**11**	39
*Sesamum indicum*	Sesame	Pedaliaceae	2	0	0	0	1	0	0	**8**	0	0	0	1	12
*Sida acuta*	Wireweed	Malvaceae	0	**3**	**3**	0	1	2	**3**	**48**	**6**	1	1	2	70
*Solanum incanum*	Nightshade	Solanaceae	2	0	0	0	0	0	0	**5**	1	1	0	0	9
*Solanum lycopersicum*	Tomato	Solanaceae	**4**	2	0	2	1	**6**	2	**14**	1	**7**	**3**	**8**	50
*Solanum melongena*	Eggplant	Solanaceae	1	0	**5**	0	0	**3**	2	**9**	1	**10**	1	**3**	35
*Solanum nigrum*	Black nightshade	Solanaceae	0	0	0	0	0	0	2	**3**	0	0	0	**3**	8
*Vigna unguiculate*	Cowpea	Fabaceae	**4**	1	0	0	0	0	0	0	0	0	0	0	6

Plant associations supported by at least 3 whiteflies were selected from Table 1 and are represented in bold. Abbreviations for the whitefly species: SSA = sub-Saharan Africa, SG = subgroup, MED = Mediterranean, ASL = African silver leafing, MEAM = Middle East-Asia Minor, IO = Indian Ocean, EA1 = East Africa 1 and B. Uganda = Bemisia Uganda. Total wfs refers to total number of whiteflies sampled for the plant species

Within SSA1, ‘subgroups’ have been described based on ~ 1–1.5% *mtCO1* nucleotide sequence differences (Legg et al. 2014b). Three of these subgroups have been confirmed recently to represent two distinct species, namely SSA1-SG1/SG2 as one species and SSA1-SG3 as another (Mugerwa et al. 2020). Differences in the abundance and host range of these two species are apparent in this study with the majority of one species (SSA1-SG1 and SSA1-SG2) collected from cassava, in contrast to none of 29 whiteflies typed as SSA1-SG3. The greatest numbers of SSA1-SG3 were from eggplant (5/29 SG3 sequences) and common bean (5/29 SG3 sequences) (Table 1). For the other SSA species collected (SSA6, SSA9-SSA16), there was also no association with cassava, with the only clear pattern of association being that of SSA6 with African basil (70/81 SSA6). For the other SSA species, five of them (SSA11, SSA12, SSA14-SSA16) were only detected once or twice and hence host associations could not be inferred. For SSA9, 27 whiteflies were distributed across eight plant families, but with the greatest number (6/27) on tomato (Table 1). No host associations were visible for either the nine SSA10 whiteflies or the 24 SSA13 whiteflies collected in this study.

Six of the plant species (bean, eggplant, pumpkin, tomato, *S. acuta* and African basil) from which ≥ 3 whiteflies of SSA1 were collected, also represented plants from which MED-ASL was collected. The latter species appears the most polyphagous of the whitefly species sampled in this study, with ≥ 3 whiteflies also collected from various brassicas, okra, sesame, sweet potato, as well as weeds including *Aspilia africana, Hoslundia opposita* and *Solanum incanum*. The predominant sampled plants for MED-ASL in this study were pumpkin (20.6%), *S. acuta* (18.1%) and sweet potato (14.3%) (Table 1). Whiteflies belonging to the globally distributed and highly polyphagous MED-Q1 and MEAM1 species were much less abundant than MED-ASL, with only 47 MED-Q1 and 39 MEAM1 whiteflies detected in contrast to the 265 MED-ASL. MED-Q1 were collected predominantly from tobacco (26/47), whereas the largest proportions of MEAM1 flies came from eggplant (10/39) and tomato (7/39).

Within the Uganda HLGG clade, B. Uganda1 was the most abundant (*n* = 105) with ≥ 3 whiteflies collected from bean, *Bidens pilosa, Conyza sumatrensis*, eggplant, *Solanum nigrum*, sweet potato, tobacco and tomato. The highest occurrence of B. Uganda1 was on sweet potato (37/105), common bean (11/105) followed by tomato (8/105). For the other ‘Bemisia Uganda’ whiteflies, only one whitefly was found for each of B. Uganda2 and B. Uganda5 species (collected from bean and *Dichrocephala integrifolia*). Four whiteflies of B. Uganda3 were detected (from bean and two weeds) and two of B. Uganda4 (from sweet potato and a weed) (Table 2).

The three plant species (sweet potato, bean and tomato) that B. Uganda1 was collected in highest numbers from were the same from which high numbers of *B. tabaci* species were collected (Table 4); the following plant species were ones on which the most diverse number of *Bemisia* species were found: bean (SSA1, MED-ASL, B. Uganda1), eggplant (SSA1, SSA9, MED-ASL, MEAM1, B. Uganda1), pumpkin (SSA1, SSA9, MED-ASL), sweet potato (MED-ASL, B. Uganda1), tomato (SSA1, SSA9, MED-ASL, MEAM1, IO, B. Uganda1), *Sida acuta* (SSA1, SSA13, MED-ASL, MED-Q1) and African basil (SSA1, SSA6, MED-ASL).

### Geographical distribution of prevalent whitefly species

**Fig. 4:**
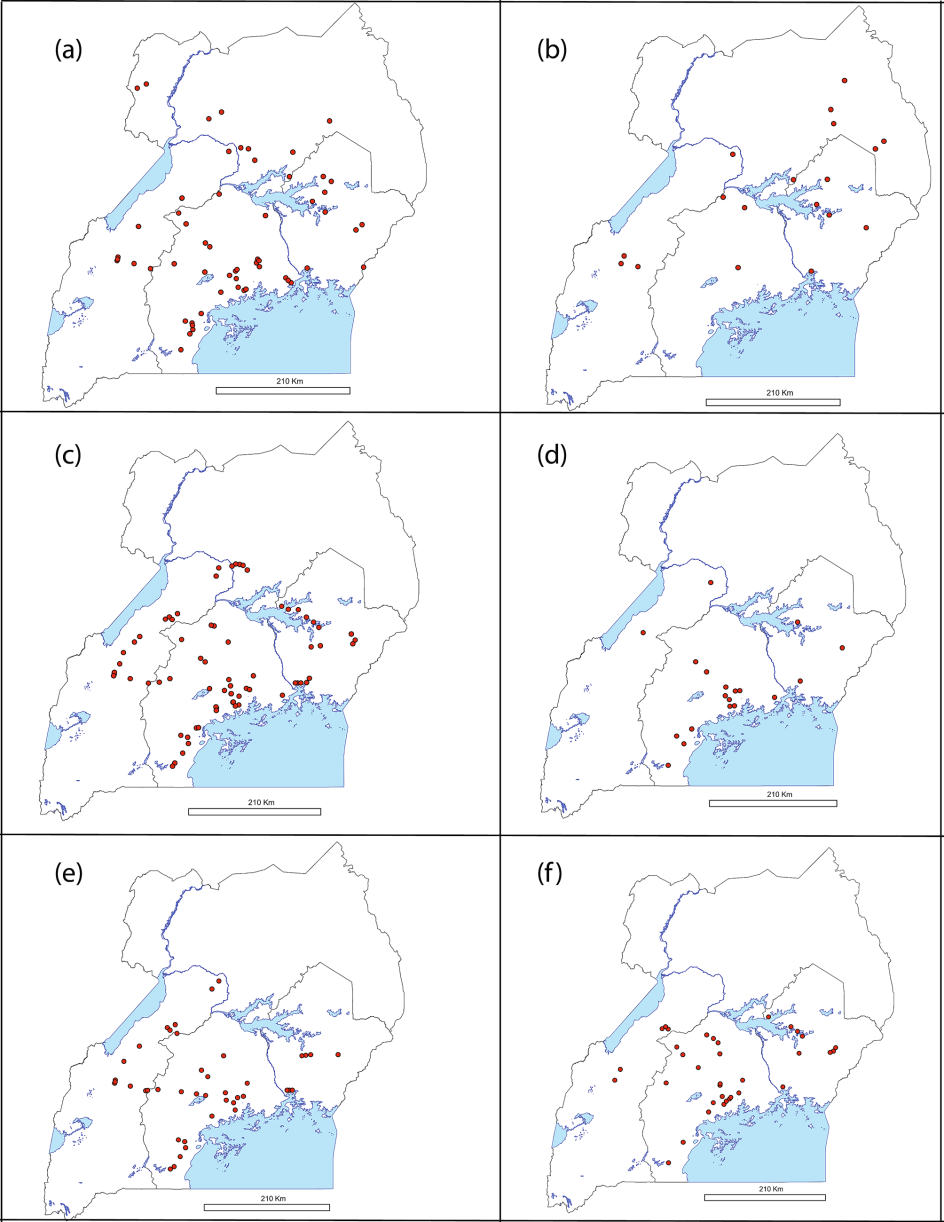
Sampled locations (red dots) for whitefly specimens in Uganda used in genetic analysis. Geographical distribution of **a** SSA1-SG1, **b** SSA2, **c** MED-ASL, **d** MED-Q1, **e** Bemisia Uganda1 and **f** SSA6 species collected during July–August 2013. Abbreviations for the *B. tabaci* species: SSA = sub-Saharan Africa (SG = subgroup), MED = Mediterranean and ASL = Africa silver leafing

Figure 4 details the geographical distributions of the most predominant *B. tabaci* species (MED-ASL 30.5%, SSA1 22.7%, SSA6 9.3%, MED-Q1 5.4%, MEAM1 4.5% and SSA2 4.1%). SSA1(-SG1) occurred throughout the country (Fig. 4a), mostly in the central region (80/137), followed by the northern (24/137), eastern (17/137) and western (16/137) regions. SSA1-SG2 was collected only in the central region (32/32), the same region from which the highest number of SSA1-SG3 whiteflies was collected from 18/29. SSA1-SG3 was also collected from the northern region (9/29) and occasionally in the western (2/29) region. SSA2 was prevalent in the northern region (22/36) and occurred less in the eastern (8/36), western (4/36) and central (2/36) regions (Fig. 4b). This contrasts markedly with SSA1 as well as all other species detected which were in lower prevalence in northern Uganda. The MED-ASL population occurred throughout the country and mostly in the central region (99/265), followed by the eastern (85/265) and western (63/265) regions. It occurred least in the northern region (18/265) (Fig. 4c). The other MED population identified, namely Q1, occurred only in the central (39/47), eastern (13/47) and western (5/47) regions (Fig. 4d). The B. Uganda1 species was also found abundant (12.1% of sequences) in this survey in all regions of Uganda except the north (Fig. 4e). It occurred most in the central region (55/105), followed by the western region (37/105) and least in the eastern region (13/105). Like B. Uganda1, SSA6 had a countrywide distribution except for the north (Fig. 4f). It occurred most in the central region (44/81), followed by the eastern (19/81) and western (18/81) regions. The MEAM1 species occurred in areas close to Lake Victoria in the central region (figure not shown).

## Discussion

### Whitefly genetic diversity

This study represents one of the most comprehensive studies in Africa to date to establish the identity of whiteflies on key crops and neighbouring weeds. Twenty-one whitefly species were identified through their partial *mtCO1* sequences, of which seven represented novel species diverging by > 4% with any *mtCO1* nucleotide sequence in GenBank. Three of these seven clustered within the *B. tabaci* clade and are named as *B. tabaci* SSA14, SSA15 and SSA16, following the proposed nomenclature by Boykin et al. (2018). The four other new whitefly species grouped outside the *B. tabaci* species complex cluster (sharing only 86.9–88.1% maximum nt identity with GenBank sequence—PDBI–MN056066) and were provisionally named B. Uganda2–B. Uganda5 prior to more thorough taxonomic classification. The remaining 14 whitefly species substantiated earlier reports from East Africa of whiteflies classified as B. Uganda1 and 13 *B. tabaci* species (Sseruwagi et al. 2005; Legg et al. 2014a, b; Mugerwa et al. 2018). The high genetic diversity observed in whitefly in Uganda, compared with the rest of the world adds further support to East Africa being a centre of origin of *B. tabaci* (Mugerwa et al. 2018).

The discovery of a further seven novel *Bemisia* species in this study was unexpected as considerable whitefly molecular characterisation studies have been carried out in SSA in the past two decades focussed on sampling from cassava, with inclusion of a few other crop plants and weeds (Burban et al. 1992; Brown et al. 1995, 2000; Legg et al. 2002, 2014a, b; Abdullahi et al. 2003; Sseruwagi et al. 2005, 2006; Mugerwa et al. 2012, 2018; Esterhuizen et al. 2013). The probable reasons for the increased diversity detected are considered the expansion of the collections to previously unsampled plant species, the use of an improved diagnostic primer set (Mugerwa et al. 2018) and the increased number of whiteflies characterised. All seven of the new species were present in only low numbers (1–4 whiteflies). The use of three whiteflies per sample was also critical in obtaining a clearer indication of whitefly-plant associations, with around 40% of samples containing multiple whitefly species per crop location as also reported previously (Gnankine et al. 2013). For some samples, the three whiteflies sampled were all different, e.g. a single *Dichrocephala integrifolia* weed sample was found to contain a mix of SSA1, MED and B. Uganda5.

### Abundance and host range of whitefly species

The most prevalent whiteflies were classified as *B. tabaci* MED-ASL (30.5% samples), SSA1 (22.7%) and B.Uganda1 (12.1%). These species were also indicated to be the most polyphagous occurring on 33, 40 and 25 identified plant species, respectively. It should be noted that for many of these plant species only a single whitefly was collected, and hence, associations with feeding on these plant species cannot be made. The survey performed focussed on gaining an idea of the potential host range of abundant whitefly populations in Uganda, and further sampling is necessary at multiple times throughout cropping seasons to validate associations. To confirm whitefly colonisation, follow-on surveys should examine nymphal development stages (instars) on the identified plant species, as performed by Sseruwagi et al. (2006). The presence of either eggs or adults on a plant is not necessarily linked to colonisation of the host, as demonstrated by Vyskočilová et al. (2019); for example, MED-ASL laid twice as many eggs on bean versus cotton, but development to adulthood was over 20-fold higher on cotton than bean. Furthermore, for whiteflies to play an important role in vectoring a plant virus, there is no need for colonisation with the efficiency of transmission dependent on feeding behaviour and whitefly virus specificity (Czosnek et al. 2017; Chi et al. 2020).

Hierarchical clustering revealed clear host preferences of *B. tabaci* species for MED-ASL (pumpkin, *S. acuta* and sweet potato), MED-Q1 (tobacco), SSA1 and SSA2 (cassava), SSA6 (African basil) and B. Uganda1 (sweet potato) assisting in predicting probable identity of whiteflies on these plant species. Specific whitefly–host associations were revealed most markedly for cassava where all 98 whiteflies were SSA1-SSA2 supporting previous reports (Legg 1996; Sseruwagi et al. 2006; MacFadyen et al., 2020). It is noteworthy that even for the most abundant non-cassava populations, namely MED-ASL (*n* = 265) and B. Uganda1 (*n* = 105), not a single whitefly was collected from cassava. The presence of a single whitefly on a plant species for much less prevalent species therefore might indicate a potential host. Support for this comes from a number of examples in the data. For SSA1-SG1, only 1/137 whiteflies characterised came from eggplant (n = 38) which would normally be considered biologically insignificant, yet eggplant is a common host plant for rearing *B. tabaci* populations including SSA1-SG1 in many insectaries (Lisha et al. 2003; Shah and Liu 2013; Vyskočilová et al. 2019). Similarly, SSA1-SG3 whiteflies were shown by Milenovic et al. (2019) to feed successfully on not only cassava but also on sweet potato and tomato. In this study, these plants would not have been indicated to be potential hosts with either none or only a single SSA1-SG3 whitefly found to be present. The presence of large numbers of SSA1 flies on common bean (17/41) and cowpea (5/6) is therefore tentatively considered as an indication of these being alternate hosts, recently verified for SSA1 on cowpea by Macfadyen et al. (2020). Furthermore, if plant hosts from which ≥ 3 whiteflies are considered as reliable indicators, this study suggests pumpkin, tomato, eggplant, as well as the weeds *Erythrina abyssinica* and *S. acuta* are additional possible alternate hosts for SSA1 ‘cassava’ whiteflies.

The most prevalent species MED-ASL in this survey was associated with high whitefly populations observed on some crops e.g. pumpkin (55/70 pumpkin whiteflies), sweet potato (38/79) and tomato (14/51) but not at all to cassava (0/98). The strong association with sweet potato in the field has been noted previously (Sseruwagi et al. 2006; Misaka et al. 2019), and recent studies have verified that sweet potato is a preferred host for MED-ASL under laboratory as well as field conditions (Vyskočilová et al. 2019; Macfadyen et al. 2020). These data support the proposal of Vyskočilová et al. (2018, 2019) to classify MED-ASL as a distinct species from MED-Q1, due to these populations failing to interbreed, showing a distinct *mtCO1* phylogenetic placement, as well as marked differences in their preferred host ranges. The limited distribution and association of MED-Q1 with tobacco was noted previously by Sseruwagi et al. (2006), but at the time it was considered possibly to be due to insufficient sampling. MED-Q1′s dominance on tobacco (26/35 tobacco whiteflies sampled) in contrast to only one MED-ASL whitefly from tobacco corroborates the insectary studies of these two MED populations on tobacco, where it was a suitable host for MED-Q1 but lethal for MED-ASL (Vyskočilová et al. 2019).

Whiteflies belonging to the globally invasive MED-Q1 and MEAM1 species (Brown et al. 1995; Liu et al. 2007; De Barro et al. 2011) were much less abundant than MED-ASL in this study, with only 47 MED-Q1 and 39 MEAM1 whiteflies detected. It is surprising that in Uganda, MEAM1 and MED-Q1 have not displaced indigenous populations, as has been the norm globally (Brown et al. 1995; Moya et al. 2001; Liu et al. 2007). A similar prevalence of MED-ASL versus MED-Q1 and MEAM1 in Uganda was reported by Sseruwagi et al. (2005) in 2003/04, a decade before the present survey was conducted and acting as confirmation that neither MEAM1 or MED-Q1 are recent introductions as the low numbers recorded in the present study might attest. Elsewhere, the success of MED populations has been associated with insecticide resistance in MED populations (Sun et al. 2013, 2014). Although the degree of insecticide resistance in MED-ASL is unknown, this is considered an unlikely explanation as insecticide usage in the smallholder farm plots sampled in Uganda was generally limited. Therefore, the factors behind MEAM1 and MED-Q1′s low occurrence in Uganda compared to MED-ASL are unclear, but may be linked to agroecology with clear plant host range differences between these three populations.

Other species that classify into the Africa-Middle East-Asia Minor cluster (Dinsdale et al. 2010) add support to this high-level genetic group (HLGG) representing polyphagous member species of the *B. tabaci* complex (Brown et al. 1995; Liu et al. 2007; De Barro et al. 2011; Malka et al. 2018, 2020). SSA12 and SSA13 are recently discovered species (Mugerwa et al. 2018), and little information is yet available on their host range. SSA12 was only collected from two unidentified uncultivated plants and appears therefore to be a native whitefly population of little risk to national agricultural productivity. The collection of 24 whiteflies of SSA13 from over a dozen different plants suggests that this is a polyphagous species that has potential to pose a greater risk.

### Geographical distribution of prevalent whitefly species

This study revealed the importance of sampling as wide a geographical region as possible covering all agroecological zones. The north-east of Uganda had not been sampled for about 30 years due to political instability from the late 1980s to the early 2000s (Barkan, 2011; Arieff et al. 2015), and SSA2 whiteflies were previously thought to have more or less disappeared (Sseruwagi 2004; Mugerwa et al. 2012; Legg et al. 2014b). This study showed that SSA2 was still the most prevalent on cassava in the northern region (22/36 SSA2 samples). This is in marked contrast to four other species that were more prevalent than SSA2 on a countrywide basis, namely MED-ASL (*n* = 265) detected at low frequency, and SSA6 (*n* = 81), MED-Q1 (*n* = 47) and B. Uganda1 (*n* = 105) that were not detected at all in northern Uganda in this study (Fig. 4). MacFadyen et al. (2020) also found SSA2 to be present in only a few regions of central to northern Uganda, and in neighbouring South Sudan, SSA2 was reported recently as the most prevalent (Misaka et al. 2019) adding weight to the prevalence and locality of SSA2 being linked to agroecology and landscape factors rather than a chance event caused by the timing of our survey.

SSA1(-SG1) is prevalent across the rest of Uganda and this is considered linked to its ability to feed on and colonise multiple host plants including cassava (Sseruwagi et al. 2006; Milenovic et al. 2019). In northern Uganda, SSA1-SG1 is prevalent on cassava in distinct regions from SSA2. As ‘superabundant’ populations have been associated with both SSA1-SG1 and SSA2 (Legg and Ogwal 1998; Legg et al. 2002, 2014b; Sseruwagi et al. 2005; Mugerwa et al. 2012), determining the factors that influence the distribution of cassava whitefly populations (SSA1 and SSA2 species) is key for the development of effective management practices for both insect pest and vectored cassava viruses.

### Implications of diversity and abundance for control of whitefly populations

Integrated pest management (IPM) approaches to control geminivirus diseases include the use of resistant cultivars, virus- and vector-free planting material, roguing of infected plants and insect vector management (Legg et al. 2005, 2014a; Rojas et al. 2018). Recommended measures are most effective for annual crops if these can be combined with host-free periods and when designed in relation to the biology and ecology of the virus and vector and the crop. All of these recommendations are, however, hard to implement in SSA at the smallholder level with susceptible crop and weed hosts being present year-round. The wide distribution of SSA1, MED-ASL and B. Uganda1 whitefly species in the diverse agroecologies on crops as well as uncultivated plant species will enable whiteflies to be easily reintroduced to targeted control areas from neighbouring fields either by wind or on plant material moved by humans. Effective control therefore needs to focus on identifying host resistance to whiteflies to reduce the high vector populations associated with viral disease outbreaks, as well as direct feeding damage. Considerable research efforts targeting resistance to cassava whiteflies are ongoing (http://www.cassavawhitefly.org), and it is hoped these efforts will be transferable in the near future to facilitate breeding for resistance to prevalent whitefly species impacting other crops in SSA.

Application of pesticides to reduce whitefly populations on high value crops like tomato and cabbage is becoming a more common practice among African small-scale farmers (PARM 2017). Globally, application of insecticides such as neonicotinoids to control high whitefly populations on various crops has resulted in the development of insecticide resistance in *B. tabaci* (Horowitz et al. 2005; Naveen et al. 2017) with to date reports of *B. tabaci* resistance to > 60 active ingredients used in insecticides (www.pesticideresistance.org). The development of insecticide resistance is generally delayed by the presence of refuge plants, but for haplodiploid pests such as *B. tabaci*, simulation studies have projected that there should be no significant effect on the evolution of resistance (Crowder et al. 2009). In the Ugandan farming system, this study has illustrated the presence of a wide variety of alternate hosts for the prevalent SSA1(-SG1), MED(-ASL) and B. Uganda1 colonising the high value crops. Although the impact of these refuges on the development of insecticide resistance is not known, it is clear that they enable reintroductions of the whitefly population(s) to occur shortly after the pesticides have lost their efficacy in the target crop plants. Moreover, in time with repeated use to control high whitefly populations on high value crops, selection will operate for insecticide resistance. This may favour the emergence of the MED-Q1 species, currently appearing to be restricted to tobacco, as globally this has developed rapid resistance to insecticides after their use (Horowitz et al. 2005; Roditakis et al. 2009; Dennehy et al. 2010). Particular care needs to be taken to try to avoid the development of insecticide resistance in MED-ASL considering its prevalence and polyphagous nature.

### Implications of diversity and host range for control of whitefly-vectored viruses

Rey et al. (2012) have reviewed the emergence of begomovirus disease outbreaks on the African continent and proximal Indian Ocean islands. They concluded that the emergence of begomovirus disease outbreaks is likely to be due to introduction and intensive cultivation of exotic crop species having been introduced into environments harbouring indigenous begomoviruses. Uncultivated wild plants were suggested to be original hosts for many of the causal viruses from which ‘spillover’ to crops has occurred, being enabled by the presence of polyphagous whitefly vector populations (García-Arenal and Zerbini 2019). Alternate host plants are known to act as reservoirs of cassava viruses and inoculum diversity (Ndunguru et al. 2005; Alabi et al. 2008; Amisse et al. 2019). The wide occurrence of some whitefly species [SSA1 (-SG1), MED (-ASL) and *B*. Uganda1] on both agricultural and weed plant species could potentially increase the acquisition and transmission of begomoviruses between plants hence resulting in mixed infections. Recombinant begomoviruses are commonly detected in cassava (e.g. Zhou et al. 1997; Berrie et al. 2001; Maruthi et al. 2002), and their origin often involves viruses from other hosts indicating how the polyphagous nature of the vector population can facilitate the creation of novel viruses.

## Conclusions

An extensive countrywide survey of whiteflies in Uganda revealed 16 *B. tabaci* (three novel) and five closely related species (four novel) present in 870 whiteflies characterised from a total of 84 different plant species. The three most prevalent whitefly species, MED-ASL, SSA1 and B. Uganda1, together accounted for ~ 65.3% of all the whiteflies. These whitefly species were also indicated by their presence on numerous plant species to be the most polyphagous. Samples of the exotic crops bean, tomato, eggplant and pumpkin, and uncultivated plants *S. acuta* and African basil possessed the greatest diversity of whitefly species. These plant hosts coincide with those known globally to contain a wide diversity of recombinant begomoviruses. All whitefly species collected from crops were also found on uncultivated plants.

The knowledge generated in this study of potential alternate hosts for the different whitefly species should be borne in mind when devising management strategies for these important agricultural pests. For each alternate host, further research will be needed to determine to what extent it contributes significantly to the population dynamics of specific whitefly species. There is currently also scant knowledge of virus variability and transmission pathways in uncultivated plant hosts and transmission to crops. Future efforts should aim at correlating the whitefly diversity observed with their roles in vectoring viruses from uncultivated plants to crops. Advances made in deep sequencing technologies and reductions in their cost are now at a stage that enable detailed geometagenomic approaches to gain a fuller understanding of vector and virus diversity and evolution driving emerging begomovirus disease outbreaks that have for several decades been threatening food security in Africa.

## Additional information

Accession numbers: the nucleotide sequences of the 870 *B. tabaci* samples used in this study were deposited in the GenBank nucleotide database, with accession codes MK444227—MK445130.

## Supplementary Information

Below is the link to the electronic supplementary material.

Supplementary file1 (PDF 69 KB)

Supplementary file2 (TIF 25677 KB)

Supplementary file3 (TIF 26360 KB)
