# Supplementary figures and images for: Genetic diversity of whitefly (Bemisia spp.) on crop and uncultivated plants in Uganda: implications for the control of this devastating pest species complex in Africa
Source: J Pest Sci (2004). 2021 Mar 10;94(4):1307–30. doi: 10.1007/s10340-021-01355-6 (PMC8550740; doi:10.1007/s10340-021-01355-6)

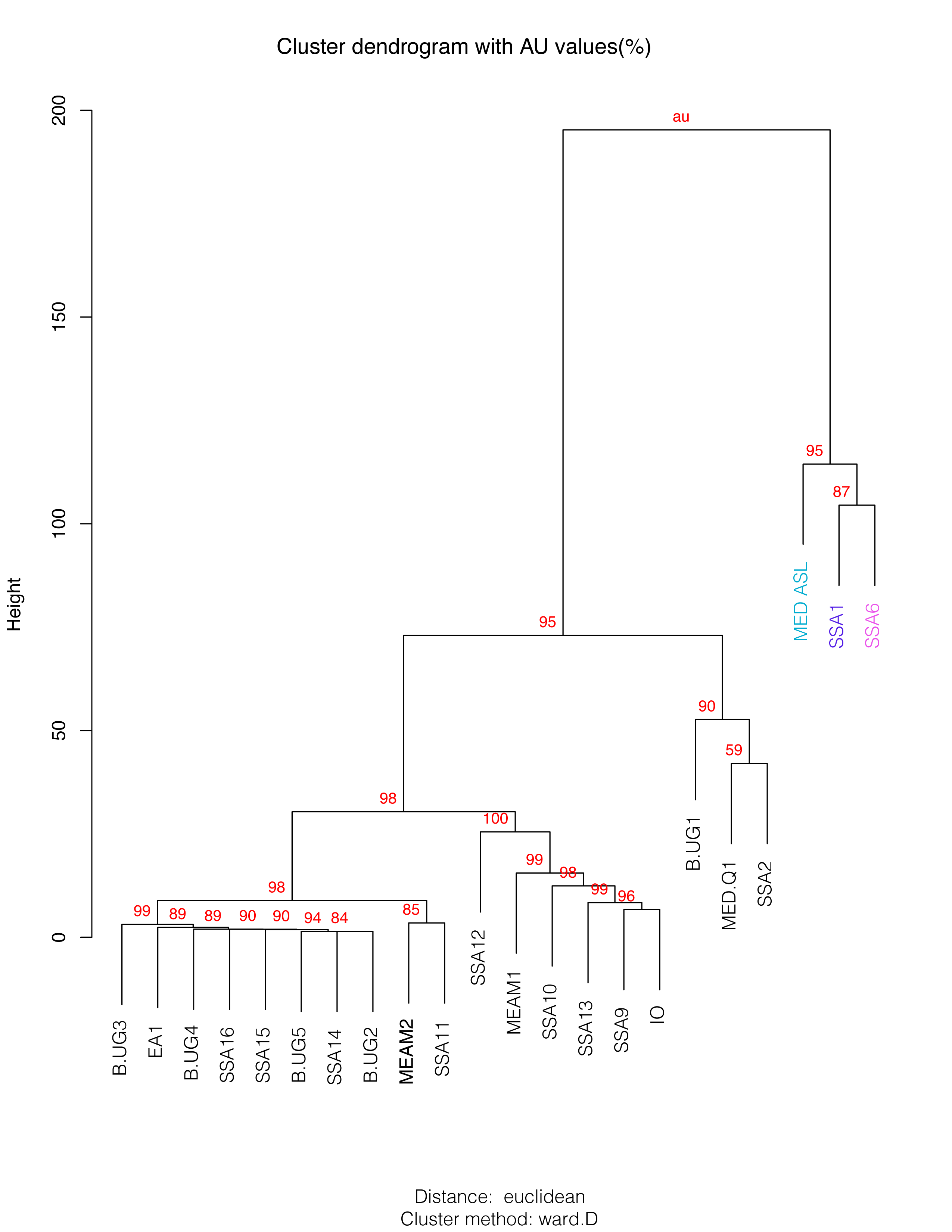

Supplement: Supplementary file 2 — Supplementary file2 (TIF 25677 KB) [file 10340_2021_1355_MOESM2_ESM.tif]

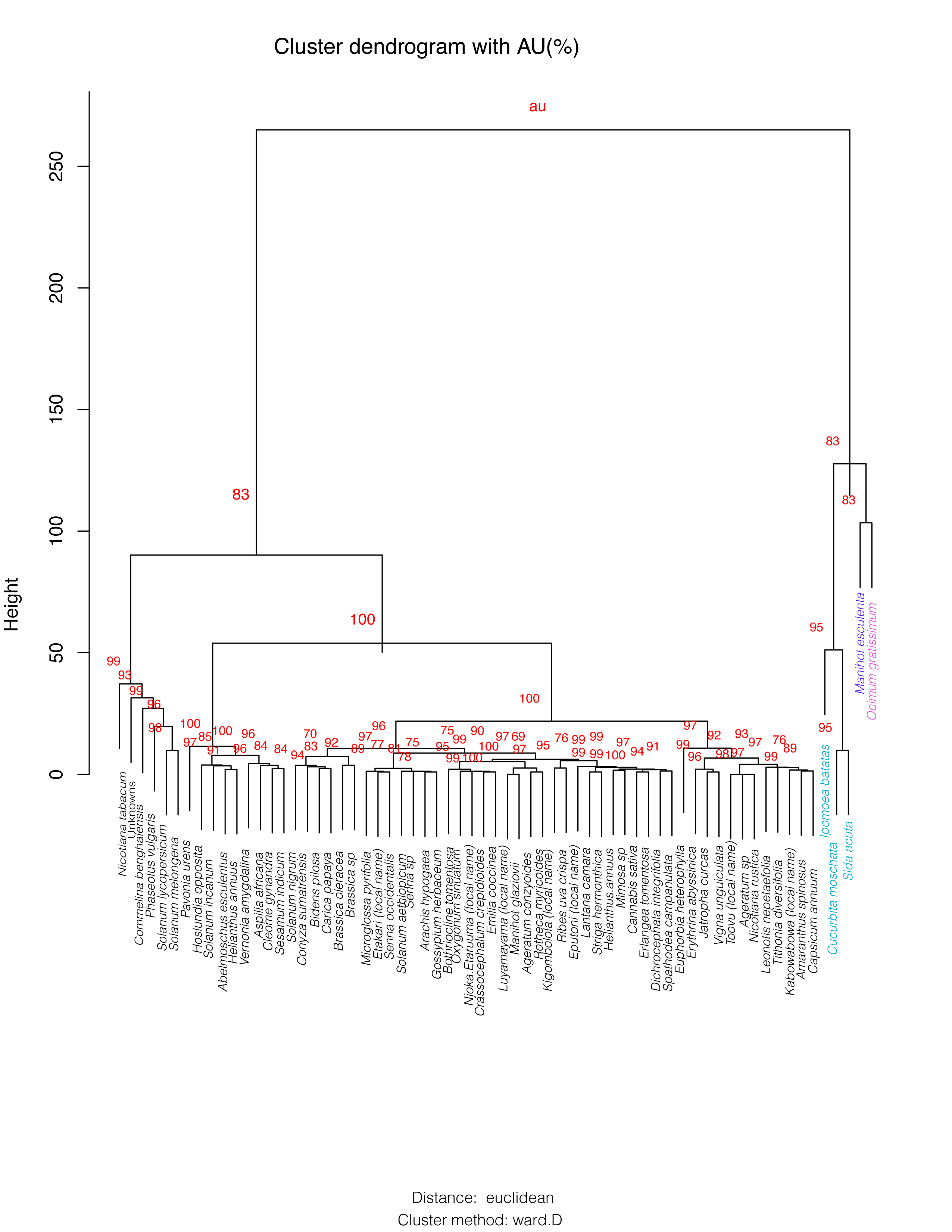

Supplement: Supplementary file 3 — Supplementary file3 (TIF 26360 KB) [file 10340_2021_1355_MOESM3_ESM.tif]
