# Supplementary material for: Genetic diversity of whitefly (Bemisia spp.) on crop and uncultivated plants in Uganda: implications for the control of this devastating pest species complex in Africa
Source: J Pest Sci (2004). 2021 Mar 10;94(4):1307–30. doi: 10.1007/s10340-021-01355-6 (PMC8550740; doi:10.1007/s10340-021-01355-6)

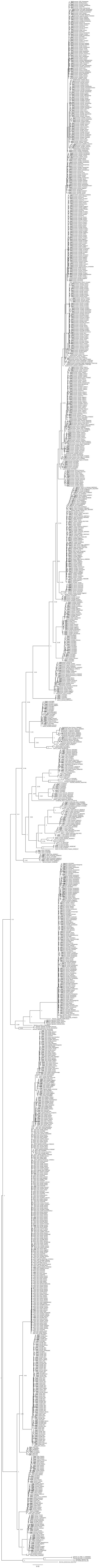

Supplement: Supplementary file 1 — Supplementary file1 (PDF 69 KB) [file 10340_2021_1355_MOESM1_ESM.pdf]
